# Uterine Smooth Muscle Tumor of Uncertain Malignant Potential (STUMP): A Case Report

**DOI:** 10.7759/cureus.103536

**Published:** 2026-02-13

**Authors:** Fernando J Interian-Alvarez, Jocelyn Pumares-Campos, Antonio Reyes-Cabrera, Brayan J Ortiz-Villanueva, Diana L Mendoza-Arcique

**Affiliations:** 1 Department of Gynecology and Obstetrics, Hospital Regional "Elvia Carrillo Puerto" Institute for Social Security and Services for State Workers (ISSSTE), Mérida, MEX; 2 Faculty of Medicine, Universidad Autónoma de Yucatán, Mérida, MEX; 3 Department of Pathology, Hospital Regional "Elvia Carrillo Puerto" Institute for Social Security and Services for State Workers (ISSSTE), Mérida, MEX; 4 Department of Medicine, La Salle University, Mexico City, MEX; 5 Department of Gynecological Endoscopic Surgery, Hospital Regional Adolfo López Mateos, Institute for Social Security and Services for State Workers (ISSSTE), Mexico City, MEX

**Keywords:** abnormal uterine bleeding, case report, histopathological diagnosis, hysterectomy, long-term surveillance, recurrence risk, stump, uterine mesenchymal tumor, uterine smooth muscle tumor of uncertain malignant potential

## Abstract

Uterine smooth muscle tumors of uncertain malignant potential (STUMP) are rare mesenchymal neoplasms that occupy a diagnostic gray zone between benign leiomyomas and malignant leiomyosarcomas. Owing to their low incidence and overlapping clinical, radiological, and histopathological features, STUMPs pose significant diagnostic and therapeutic challenges and are frequently identified only after surgical intervention for presumed benign disease. We report the case of a 39-year-old Mexican woman with a two-year history of abnormal uterine bleeding (AUB) and severe dysmenorrhea. Preoperative imaging findings were consistent with an intramural uterine mass and diffuse adenomyosis. The patient underwent a total abdominal hysterectomy with bilateral salpingectomy. Histopathological examination revealed a smooth muscle tumor characterized by focal nuclear atypia, a low mitotic index of up to 2 mitoses per 10 high-power fields, and absence of coagulative tumor cell necrosis (CTCN), findings consistent with a diagnosis of STUMP. The postoperative course was uneventful, and the patient was referred for long-term clinical surveillance. This report constitutes the first indexed case of STUMP in a Mexican patient and underscores the importance of thorough histopathological evaluation for accurate diagnosis, as well as the relevance of structured long-term follow-up given the potential for delayed recurrence.

## Introduction

Uterine smooth muscle tumors encompass a heterogeneous spectrum of neoplasms, ranging from benign leiomyomas to highly aggressive leiomyosarcomas. In response to tumors that do not meet the clear criteria for either category, the World Health Organization (WHO) updated its classification in 2020 to include smooth muscle tumor of uncertain malignant potential (STUMP) as a distinct diagnostic entity, alongside leiomyoma variants, intravenous leiomyomatosis, metastasizing leiomyoma, and leiomyosarcoma [1]. STUMP is a diagnostic gray area applied to tumors that exhibit histopathological features that exceed those of benign leiomyomas, but which are insufficient for a definitive diagnosis of leiomyosarcoma [2].

From a pathological standpoint, STUMPs are primarily defined by morphological criteria rather than clinical behavior. Their classification relies on the combined evaluation of three key histological parameters: the degree of cytologic atypia, mitotic activity, and the presence or absence of coagulative tumor cell necrosis (CTCN). Tumors demonstrating an atypical combination of these features without fulfilling established thresholds for malignancy are categorized as STUMPs [1,2]. This intrinsic diagnostic ambiguity reflects the biological heterogeneity of these tumors and underlies the uncertainty surrounding their clinical course.

From an epidemiological perspective, STUMPs are rare, accounting for only around 0.01% of women undergoing surgery for suspected uterine leiomyomas [3]. Clinically and radiologically, they are virtually indistinguishable from benign fibroids, as patients typically present with non-specific symptoms such as abnormal uterine bleeding (AUB), pelvic pain, or uterine enlargement. Consequently, preoperative suspicion is uncommon, and diagnosis is almost exclusively established through postoperative histopathological examination [4].

The biological behavior of STUMPs is unpredictable. While many cases follow a benign course, a subset demonstrates local recurrence or, less frequently, distant metastasis, often occurring years after the initial diagnosis. This unpredictable behavior has been attributed to underlying molecular and proliferative heterogeneity, which is not always captured by routine histomorphological assessment [2,4]. Consequently, STUMP poses a significant challenge in both diagnosis and postoperative management, particularly with regard to surveillance strategies and counseling patients about recurrence risk.

Due to the rarity of this condition and the lack of standardized management guidelines, detailed case reports are crucial for improving our understanding of STUMP, particularly in underrepresented populations. This case report has been written in line with the SCARE 2023 guidelines [5], and to our knowledge, it is the first documented case of uterine STUMP in a Mexican patient to be published in a scientific journal.

## Case presentation

A 39-year-old Mexican woman presented with a two-year history of abnormal uterine bleeding (AUB) and severe dysmenorrhea. Her obstetric history was significant for three prior deliveries (P3L3), all performed by cesarean section. The first cesarean section was carried out as an emergency due to eclampsia. Cervical cytology performed five months prior to presentation revealed a low-grade squamous intraepithelial lesion (LSIL) associated with high-risk human papillomavirus infection (HPV type 16). The patient also reported a 10-year history of uterine leiomyomatosis, for which she had previously declined both medical and surgical treatment.

The physical examination was unremarkable. Pelvic ultrasound revealed an enlarged uterus measuring 91 × 47 × 49 mm, with an endometrial thickness of 8.1 mm. Imaging findings were consistent with diffuse adenomyosis, characterized by increased vascularity, as well as a 17 × 17 mm posterior intramural leiomyoma, which was classified as International Federation of Gynecology and Obstetrics (FIGO) type 4 [6]. Baseline laboratory evaluation showed no abnormalities (Table 1).

**Table 1:** Laboratory findings in this patient and reference ranges MCV: mean corpuscular volume, PT: prothrombin time, INR: international normalized ratio, aPTT: activated partial thromboplastin time, TSH: thyroid-stimulating hormone

Parameter	Patient value	Reference range	Comment
Hemoglobin (g/dL)	13	12-16	Normal
Hematocrit (%)	36.7	36-46	Normal
Platelets (×10⁹/L)	300	150-400	Normal
PT (seconds)	10.7	10-14	Normal
INR	0.9	0.8-1.2	Normal
aPTT (seconds)	30	26-35	Normal
Glucose (mg/dL)	97	70-99	Normal
Creatinine (mg/dL)	0.8	0.5-1.2	Normal
TSH (µU/mL)	1.41	0.4-4.0	Normal

The patient underwent a total abdominal hysterectomy with bilateral salpingectomy and ovarian preservation. Intraoperative findings included an enlarged uterus measuring 90 × 70 × 60 mm and multiple focal endometriosis lesions located on the uterine serosa and posterior cul-de-sac, while both ovaries appeared macroscopically normal. The immediate postoperative course was uneventful, and the patient was discharged 48 hours after surgery.

**Figure 1:**
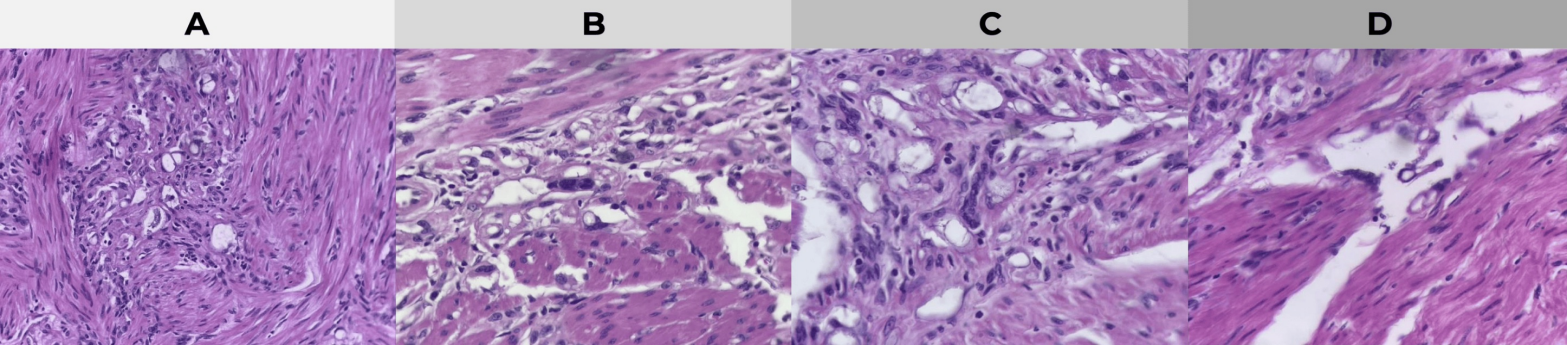
Histopathological findings of the uterine specimen (hematoxylin and eosin staining) (A) Medium-power view showing a smooth muscle tumor composed of interlacing fascicles of spindle cells with eosinophilic cytoplasm and cigar-shaped nuclei. (B) High-power magnification demonstrating focal nuclear atypia with mild nuclear enlargement and hyperchromasia. (C) Areas with cystic, vascular-like spaces displaying a sponge-like pattern within the tumor stroma. (D) High-power view confirming the absence of coagulative tumor cell necrosis, supporting the diagnosis of a spindle-cell variant of uterine STUMP according to the Stanford criteria [7]. STUMP: smooth muscle tumor of uncertain malignant potential

Histopathological examination of the surgical specimen revealed a uterine smooth muscle tumor composed of interlacing fascicles of spindle cells with eosinophilic cytoplasm and cigar-shaped nuclei (Figure 1A). High-power examination demonstrated focal nuclear atypia with mild nuclear enlargement and hyperchromasia (Figure 1B). Additional sections showed cystic, vascular-like spaces with a sponge-like pattern within the tumor stroma (Figure 1C). Importantly, no evidence of coagulative tumor cell necrosis was identified on high-power evaluation (Figure 1D), supporting the diagnosis of a spindle-cell variant of uterine smooth muscle tumor of uncertain malignant potential (STUMP) [7].

Immunohistochemical analysis was not performed because the diagnosis was established based on well-defined classical histomorphological criteria, including assessment of cytologic atypia, mitotic activity, and absence of coagulative tumor cell necrosis, which were sufficient for definitive classification. Furthermore, a fragment of vaginal mucosa showed a focal high-grade squamous intraepithelial lesion (HSIL) with negative surgical margins, and diffuse adenomyosis was also confirmed.

Postoperatively, the patient was referred to a colposcopy clinic for management of the vaginal HSIL. Given the diagnosis of STUMP, a long-term surveillance protocol was initiated, with the first follow-up scheduled at six months.

## Discussion

STUMPs represent a heterogeneous group of neoplasms that fall into a "gray zone" of uterine pathology. A systematic review published in 2022 identified 34 studies comprising a total of 189 cases of women diagnosed with STUMP. The analysis of the population characteristics revealed a median age of 43 years, a body mass index (BMI) exceeding 30 kg/m² in 41.9% of patients, and a history of parity in 69% of the cohort [8]. Although abnormal uterine bleeding (AUB) was the most frequent symptom leading patients to seek medical care (27.1%), it is noteworthy that 44.4% of the cases were incidental detections during routine gynecological evaluations [8].

Diagnosis of uterine smooth muscle tumors of uncertain malignant potential is based on the identification of classical histopathological criteria originally described by the Stanford group, including the assessment of cytologic atypia, mitotic index, and the presence or absence of coagulative tumor cell necrosis (CTCN) [9-12]. Three distinct histological variants of STUMP are recognized: spindle-cell (fusocellular), epithelioid, and mixed. In this case, the presence of focal atypia and a low mitotic index (2-4 mitoses) without necrosis allowed for the classification of a spindle-cell STUMP variant. This specific subtype accounts for approximately 12%-17% of cases and is generally associated with a lower rate of recurrence compared to other variants [8]. While immunohistochemical markers such as p16, p53, and Ki-67 can aid in risk stratification, where diffuse p16 expression and abnormal p53 staining suggest higher recurrence risks, the diagnosis remains primarily morphological [4,9].

Regarding the differential diagnosis, an adenomatoid tumor was considered due to the microscopic presence of vascular cystic spaces with a sponge-like pattern. While these benign neoplasms of mesothelial origin typically lack atypia, mitosis, or necrosis [10], the limitation of not having immunohistochemical results in this case prevented the absolute confirmation or exclusion of a coexisting adenomatoid component.

Regarding management and prognosis, the standard treatment for patients who have completed childbearing is total hysterectomy [3,4,8]. In contrast, myomectomy may be considered for patients desiring fertility preservation, although this approach requires stringent counseling regarding the risk of recurrence [4]. Available evidence indicates that unprotected morcellation represents a significant independent risk factor for recurrence and should therefore be avoided [4,8].

The recurrence rate for STUMP varies between 7.3% and 21.5%. In a systematic review of 189 cases, 37 patients (19.5%) experienced disease recurrence. Local recurrence (62.2%) involving the uterus and pelvis was found to be significantly more frequent than distant recurrence (37.8%), which most commonly affected the lungs and abdominal organs [8]. Given the lack of standardized guidelines, we adopted a protocol of semi-annual follow-up for five years, followed by annual visits for an additional five years, including pelvic ultrasound and annual thoraco-abdomino-pelvic CT scans [3].

## Conclusions

Uterine STUMP is a diagnosis of exclusion that poses a clinical challenge as it mimics symptomatic leiomyomas. In this Mexican patient, morphological criteria (focal atypia, low mitotic index, and absence of necrosis) were sufficient to confirm STUMP and rule out high-grade leiomyosarcoma despite the lack of immunohistochemical markers. The rarity of this entity and its documented potential for recurrence justify a long-term, strict surveillance plan.
